# Determining the burden of fungal infections in Zimbabwe

**DOI:** 10.1038/s41598-021-92605-1

**Published:** 2021-06-24

**Authors:** Lorraine T. Pfavayi, David W. Denning, Stephen Baker, Elopy N. Sibanda, Francisca Mutapi

**Affiliations:** 1 Nuffield Department of Medicine, Centre for Tropical Medicine and Global Health, University of Oxford, Old Road Campus, Roosevelt Drive, Oxford, OX3 7LG UK; 2 Institute of Immunology and Infection Research, University of Edinburgh, Ashworth Laboratories, King’s Buildings, Charlotte Auerbach Road, Edinburgh, EH9 3FL UK; 3 NIHR Global Health Research Unit Tackling Infections To Benefit Africa (TIBA), University of Edinburgh, Ashworth Laboratories, King’s Buildings, Edinburgh, UK; 4 Manchester Fungal Infection Group, The University of Manchester and Manchester Academic Health Science Centre, Manchester, UK; 5 University of Cambridge School of Clinical Medicine, Cambridge Biomedical Campus, Cambridge, CB2 0AW UK; 6 Department of Medicine, University of Cambridge School of Clinical Medicine, Cambridge Biomedical Campus, Cambridge, CB2 2QQ UK; 7 Asthma Allergy and Immunology Clinic, Twin Palms Medical Centre, Harare, Zimbabwe; 8 TIBA Zimbabwe, NIHR Global Health Research Unit Tackling Infections To Benefit Africa (TIBA), University of Edinburgh, Edinburgh, UK; 9 Department of Pathology, National University of Science and Technology (NUST) Medical School, Bulawayo, Zimbabwe

**Keywords:** Fungi, Diseases

## Abstract

Zimbabwe currently faces several healthcare challenges, most notably HIV and associated infections including tuberculosis (TB), malaria and recently outbreaks of cholera, typhoid fever and COVID-19. Fungal infections, which are also a major public health threat, receive considerably less attention. Consequently, there is dearth of data regarding the burden of fungal diseases in the country. We estimated the burden of fungal diseases in Zimbabwe based on published literature and ‘at-risk’ populations (HIV/AIDS patients, survivors of pulmonary TB, cancer, chronic obstructive pulmonary disease, asthma and patients receiving critical care) using previously described methods. Where there was no data for Zimbabwe, regional, or international data was used. Our study revealed that approximately 14.9% of Zimbabweans suffer from fungal infections annually, with 80% having tinea capitis. The annual incidence of cryptococcal meningitis and *Pneumocystis jirovecii* pneumonia in HIV/AIDS were estimated at 41/100,000 and 63/100,000, respectively. The estimated prevalence of recurrent vulvovaginal candidiasis (RVVC) was 2,739/100,000. The estimated burden of fungal diseases in Zimbabwe is high in comparison to other African countries, highlighting the urgent need for increased awareness and surveillance to improve diagnosis and management.

## Introduction

Africa has an estimated population of 1.3 billion people and accounts for about 75% of all the 38 million human immunodeficiency virus (HIV)-infected people in the world. Notably, approximately 50% of all fungal-related deaths due to HIV infections are thought to occur in Africa; however accurate data are lacking^1,2^. Data generated by the Global Action Funds for Fungal Infections (GAFFI), suggests an estimated 47.6 million Africans suffer from fungal diseases, of which 1.7 million suffer annually from a serious fungal infection^3^. However, these estimates are based on data from only a few African countries, and most likely underestimates the true prevalence.

Fungal diseases are life-threatening and are responsible for a largely silent epidemic, often hidden killers causing substantial morbidity and mortality in susceptible individuals^4^. Patients with fungal infections occur across a huge spectrum of medical conditions often as co-infections or opportunistic infections^5^ and are thus treated as separate entities, hindering progress in diagnosis and management of these patients^6^. Only skin, hair, nails and mucosal infections can be clinically diagnosed (with much imprecision) without specific laboratory testing or medical assessment (radiology, mycology, histopathology) with expensive technologies requiring trained personnel. On the other hand, most life-threatening infections require the referred methods to be diagnosed, which is often out of the reach of patients in poor resource settings.

Nonetheless, substantial progress is being made to prevent and manage some of these fungal diseases. Mycetoma and chromoblastomycosis have been included in the World Health Organisation (WHO) list of neglected tropical diseases^7,8^ and new guidelines for the prevention and management of cryptococcal meningitis were recently issued^3^.

Fungal infections such as histoplasmosis, mycetoma, chromoblastomycosis, sporotrichosis, cryptococcal meningitis and tinea capitis^9–13^ have been reported in Zimbabwe, albeit in few and dated reports. Therefore, there is need to update the information on the burden of these infections in Zimbabwe if they are to be prioritised for health intervention.

In the last three decades Zimbabwe’s health system has faced considerable issues^14^, most notably the demand for providing healthcare services for the control of HIV and associated tuberculosis (TB) as well as for other endemic infections such as malaria and schistosomiasis. The recent cholera^15^ and typhoid fever^16^ outbreaks further exacerbated Zimbabwe’s health challenges. The impact of the current severe acute respiratory syndrome coronavirus (SARS-CoV-2) pandemic^17^ on the health system has yet to be fully realised. Thus, given the current health prioritisations in the context of limited resources, controlling fungal infections is currently not a national priority^6^. This may only change with the quantification of the burden of these diseases and their impact on human health in Zimbabwean population.

Thus, in this study, we sought to provide estimates of the burden of fungal infections by using local published data; for those diseases with no existing local data, we used data from neighbouring countries, or international sources.

## Results

**Figure 1:**
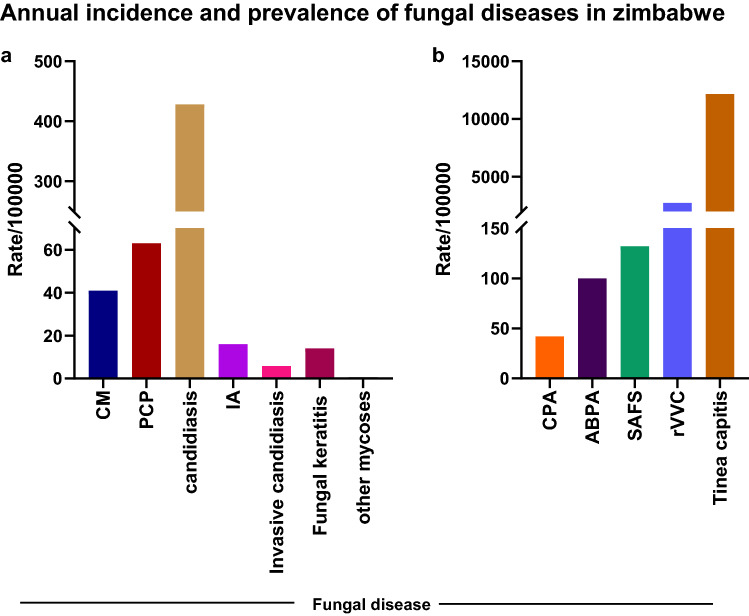
Annual incidence and prevalence of fungal infections in Zimbabwe. Bar charts representing the burden of fungal diseases per 100,000 people (**a**) incidence and (**b**) prevalence for each fungal disease with data available. CM, Cryptococcal meningitis; PCP, *Pneumocystis* pneumonia; candidiasis (oesophageal candidiasis) ; Invasive candidiasis (candidaemia and *Candida* peritonitis); IA, Invasive aspergillosis; other mycoses (histoplasmosis and mucormycosis); CPA, Chronic pulmonary aspergillosis; ABPA, Allergic bronchopulmonary aspergillosis; SAFS, Severe asthma with fungal sensitisation; rVVC, recurrent vulvovaginal candidiasis.

Using previously described methods, we were able to estimate the occurrence of 2,212,715 cases of fungal infections each year in Zimbabwe (Table 1). The rate of each fungal disease per 100,000 people in Zimbabwe is also represented in Fig. 1.

**Table 1:** Estimated burden of fungal diseases in Zimbabwe.

Number of Infections per underlying disorder per year								
Infection	None	HIV/AIDS	Respiratory disease	Cancer	Critical care + surgery	Totals	Burden	Rate/100,000
Cryptococcal meningitis		6086				6086	I	41
*Pneumocystis* pneumonia		9429				9429	I	63
Invasive aspergillosis		800	19	46	1582	2448	I	16
CPA			6182			6182	P	42
ABPA			14,892			14,892	P	100
SAFS			19,657			19,657	P	132
Candidaemia				520	223	743	I	5.0
*Candida* peritonitis					111	111	I	0.8
Oral candidiasis		77,143				77,143	I	519
Oesophageal candidiasis		63,571				63,571	I	427
RVVC (≥ 4x/year)	203,585					203,585	P	2739^a^
Mucormycosis				30		30	I	0.2
Histoplasmosis		57				57	I	0.4
Fungal keratitis	2081					2081	I	14
Tinea capitis	1,806,700					1,806,700	P	12,156
Total burden estimated	2,012,366	157,086	40,750	596	1916	2,212,715		16,255.4

*I* Incidence; *P* Prevalence; *ABPA* Allergic bronchopulmonary aspergillosis; *SAFS* Severe asthma with fungal sensitisation; *CPA* Chronic pulmonary aspergillosis; *RVVC* Recurrent Vulvovaginal Candidiasis

^a^Rate among all females.

### HIV-related fungal infections

*Cryptococcus neoformans* complex*, Pneumocystis jirovecii* (previously *Pneumocystis carinii*) and oropharyngeal candidiasis are the fungal diseases most commonly associated with AIDS. According to the UNAIDS 2019 report, 1.4 million Zimbabweans were living with HIV, 85% were on ART and 42,857 new AIDS cases at risk of opportunistic fungal infections^18^. *C. neoformans* complex is the most common cause of meningitis globally and is a leading cause of mortality among HIV-infected adults^11,19,20^ in these patients. We estimated 6,086 cases (40/100,000) of cryptococcal meningitis. PCP is a major cause of infection in those with HIV/AIDS, and unfortunately, most of these patients are undiagnosed or diagnosed late, particularly in resource-limited settings^21,22^. In Zimbabwe, the largest reported series was 8 (22%) cases of PCP in 1989 of HIV-infected individuals with respiratory symptoms^23^. Assuming 11% of newly diagnosed HIV/AIDS adults^24^, develop PCP over 2 years, we estimated 9429 cases (63/100,000) of PCP. PCP may be proportionately more common in children with HIV and was likely a significant contributor to the 3000 children who died of AIDS in 2019^18^, but we did not estimate this separately due to the absence of data. We estimated oral candidiasis to affect 77,143 individuals and oesophageal candidiasis 63,571 people living with HIV (PLHIV).

### Invasive aspergillosis (IA) and mucormycosis

We estimated a total of 2448 cases of IA annually (16/100,000) including 45 cases in haematological malignancy, 19 cases among those with lung cancer, 800 cases among people who died HIV/AIDS and 1582 cases among persons admitted to hospital with COPD. The recognized association with transplantation procedures could not be estimated since these procedures were not undertaken in Zimbabwe during the relevant period. For mucormycosis we conservatively estimated only 30 cases^25^.

### Histoplasmosis

Histoplasmosis is poorly described in Zimbabwe with limited epidemiological data. However, a study by Oladele et al.^26^ reports that *Histoplasmosis capsulatum* var *capsulatum* (HCC) and *Histoplasmosis capsulatum* var *duboisii* (HCD) coexist in Zimbabwe. We estimate 57 cases of histoplasmosis per year. This estimate excluded non-disseminated forms of histoplasmosis.

### Non-HIV-related fungal disease burden

Chronic pulmonary aspergillosis (CPA) is a complication of pulmonary TB that is often diagnosed late and may mimic pulmonary TB. It also affects patients with other pulmonary disorders, notably COPD, after pneumothorax and occasionally those with ABPA and asthma^27^. We estimated 6840 of CPA cases per year. Fungal allergy exacerbates asthma, especially in adults. The prevalence of asthma in adults in Zimbabwe was estimated to be 6.9% using data from Democratic Republic of the Congo^28^. We estimated 14,892 and 19,657 cases per year of ABPA and SAFS respectively. There may be some duplication between these entities as many ABPA patients have severe asthma. Therefore, the true ‘fungal asthma’ prevalence may be 75% of their total. Cystic fibrosis has not reported from Zimbabwe.

Candidaemia and *Candida* peritonitis were estimated to affect 743 and 111 patients, respectively. We did not estimate *Candida* peritonitis complicating chronic ambulatory peritoneal dialysis.

RVVC is defined as four or more episodes of vulvovaginal candidiasis per year^29^. We estimated 203,585 RVVC to occur among adult women in the general healthy female population in their fertile years, which may be conservative. Hormone replacement therapy can precipitate RVVC^30^, but we did not estimate this.

Fungal keratitis often occurs following ocular trauma from vegetable material^31,32^ and male agricultural workers are at a greater risk^33^. It often leads to blindness and a recent global estimate found a culture and microscopy positive annual incidence of 14/100,000, which translates into approximately 2080 cases in Zimbabwe. However, assuming that culture and microscopy negative cases are usually cases of fungal keratitis^34^ in high incidence areas, this number rises to approximately 2930 cases. Most of the infected eyes will go blind and some will perforate and require removal^34^.

**Figure 2:**
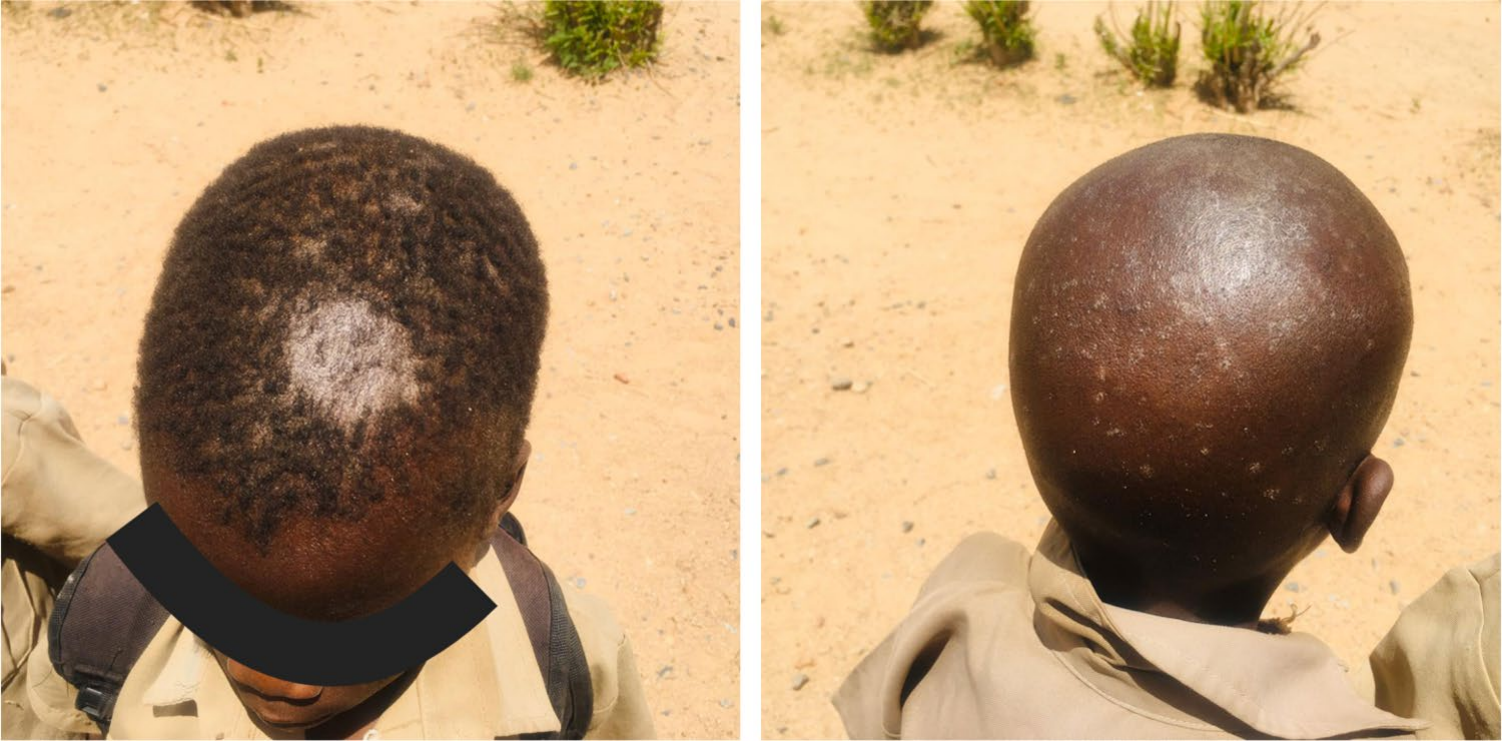
Tinea capitis infection in two young boys from rural Zimbabwe.

Cutaneous fungal infections are very common in southern Africa, but here we focus only on tinea capitis, given its transmissibility, scarring potential and occasional complications of kerion^35^. We estimated 1,806,700 schoolchildren suffering from tinea capitis. The two most frequent dermatophyte species isolated from tinea cases in Zimbabwean children were *Trichophyton violaceum* and *Microsporum audouinii*^9^. These species have been shown to be the most frequent dermatophyte species involved in tinea capitis among children in southern Africa^36–39^. This observation is in accordance with a recent systematic review estimating the burden of tinea capitis among children in Africa^40^. Figure 2 shows tinea capitis infection in two young school boys from rural Zimbabwe.

There were 14 chromoblastomycosis, 3 sporotrichosis and 5 mycetoma cases as reported by Ross and Gelfand in a 10-year survey of histological material^12,13^. Only four cases of blastomycosis were reported in 1991^41^, however some more recent isolates such as *Blastomyces gilchristii* have been described^42,43^. Given that these older estimates are the only available data in Zimbabwe, we cannot use them for a reliable estimate of the current burden. The reports however show that these conditions occur in this population and may be underdiagnosed and under reported.

## Discussion

A diverse range of fungal infections commonly occurs in Zimbabwe. Frequent HIV and TB co-infections contribute to a higher prevalence of some fungal diseases. Each of these conditions on its own or in combination can predispose individuals to fungal diseases^44^. To date, the burden of fungal infections in Zimbabwe has not been documented. Therefore, we conducted this study to estimate the burden of fungal infections/diseases in the country. Our study indicated that over 2 million people [2,212,715 (14.9%)] suffer from fungal disease annually, a higher number than most African countries. This figure is comparable to that of Senegal^45^ and Nigeria^46^ with tinea capitis being the most predominant fungal infection.

In Zimbabwe, we found that, following tinea capitis, the most frequent serious fungal diseases were recurrent vulvovaginal candidiasis, oral and oesophageal candidiasis. Vulvovaginal candidiasis (VVC) is a common gynaecological problem occurring among women globally, most commonly caused by *Candida albicans*^47,48^. In previous studies carried out in Zimbabwe among women presenting with symptomatic vaginal discharge, VVC prevalence rates ranged between 25 and 40%^49,50^ and we have estimated that 203,585 Zimbabwean women suffer from recurrent episodes. While recurrent vulvovaginal candidiasis (RVVC) is not life threatening, it is a significantly more severe clinical form than VVC. This is because of the recurrence of symptoms defined as four or more episodes per year^29,51^ and it is a major health problem for sexually active women. RVVC affects quality of life and is associated with anxiety, depression and a loss of productivity^52–54^. Our estimates, make the burden of RVVC in Zimbabwe the fifth highest among the Southern African Development Community (SADC) countries with available estimated burdens^44,55–57^ and some data indicate that the prevalence of RVCC may be higher in Africa than other continents^54,58,59^. Genetic factors have been suggested to be related to the susceptibility of Black/ African women to RVCC. However, comprehensive assessment of the role of genetics in RVCC is still lacking. Likewise, host-related and behavioural factors could also have a role to play^51,60,61^.

Oral and oesophageal candidiasis occurs commonly in AIDS patients or those with other immunosuppressive conditions. Oral candidiasis is one of the most common fungal opportunistic infections in immunocompromised individuals^62,63^ and was found to be the most common opportunistic infection in Nigeria^64^ and Uganda before the initiation of highly active antiretroviral therapy (HAART)^65^. Fluconazole is the drug of choice in the treatment of oral candidiasis^62^ because of its bioavailability and efficacy compared with other antifungal drugs^66,67^. However, an increase in resistance of *Candida* species to fluconazole has been reported in some parts of Africa^68–71^ and this is important to note because of the implications for morbidity and mortality rates^71,72^.

Cryptococcal meningitis is the leading cause of meningitis in sub-Saharan Africa^44,73^. Recent studies have shown that an increasing proportion of patients with cryptococcosis are ART-experienced^74,75^. In Uganda 3% of ART-experienced patients with virological failure were cryptococcal antigen (CrAg) positive^76^ with a background rate of 5–10% cryptococcal meningitis. Consequently, to estimate the incidence of cryptococcal meningitis in Zimbabwe we doubled our at-risk population (ART naïve), being cognisant of the fact that virological failure does not always translate to immunodeficiency. We estimated the occurrence of 6086 cases of cryptococcal meningitis per year. We estimated 9429 cases of PCP in HIV/AIDS patients only. Although, PCP is common in children^77–79^ and also occurs in non-HIV infected patients we did not include these in our estimates due to paucity of data.

The prevalence of CPA was estimated at 42/100,000, which was relatively high compared to other African countries^55–57^. South Africa had the highest prevalence, at 176/100,000^44^. The high number in Zimbabwe could reflect the relatively high burden of tuberculosis^80^ and further studies are required to validate this. The diagnosis requires a combination of imaging and *Aspergillus* IgG antibody testing recently recognised as an Essential Diagnostic by WHO^81^, however these are not routinely done in Zimbabwe. Notably a fifth (19%) of CPA patients who were TB smear negative and GeneXpert negative in Nigeria were incorrectly diagnosed as having pulmonary TB^82^ with consequent inappropriate treatment.

Asthma is a significant public health problem in Zimbabwe and is often poorly controlled^83^. Here we estimated that 595,677 adults have asthma. In Zimbabwe fungal sensitisation studies have not been conducted, nonetheless we were able to estimate the burden for ABPA and SAFS, which are collectively known as ‘fungal asthma’. Fungal asthma differs from allergic asthma. Although the bronchoconstriction can be alleviated by the bronchodilators and inhaled glucocorticosteroids used in the management of asthma, fungal diseases requires the administration of antifungal agents such as oral itraconazole^84–87^ and voriconazole, which can only be prescribed if an accurate diagnosis has been made. These antifungal agents act by reducing the fungal load, thus minimizing the stimulus for the ongoing inflammatory activity^88^. If inadequately managed fungal asthma can lead to significant complications such as long term steroid toxicity, bronchiectasis and CPA^89^.

A review on the role of antifungals in the management of patients with severe asthma has recently been published^87^. The paper highlights significant studies that confirm lower toxicity of treatment with azoles, particularly itraconazole for ABPA and provide recommendations for the use of antifungal agents in patients with severe asthma, airways fungal infection and fungal colonisation^87^.

Dermatophyte infections, especially tinea capitis, are common among children all over Africa, particularly in areas with poor socioeconomic and sanitary conditions^90–92^. They are a public health problem due to their contagious nature^55^. Prevalence rates range from 10% to more than 70% in different regions of Africa^9,40,93,94^. The estimate of tinea capitis in this study was based on Zimbabwean data from 1990^9^. However, there may have been changes over time and these figures may not be a true presentation of the current situation in the country. Nonetheless, it remains a common clinical problem.

Mycetoma is a neglected tropical disease caused by fungi or bacteria and mainly affects the skin as well as the underlying tissues^95^. The morbidity due to mycetoma is high^96^ and there are currently no control programmes except in Sudan where it is highly endemic^7,8,97^. Cases of mycetoma have been reported in many African countries^45,98–103^ including Zimbabwe^12^. Cases of chromoblastomycosis, sporotrichosis and blastomycosis have also been reported in Zimbabwe. However, to date there are no reports of disseminated *Emergomyces* infections in Zimbabwe albeit there are some reports from South Africa^104^. Contributory factors include a low index of clinical suspicion, limited diagnostic capacity and a dearth of the requisite clinical and diagnostic expertise.

Most fungal infection studies in Zimbabwe are dated having been carried out about two decades ago^9–11,23^. While these indicate susceptibility in this population, they do not accurately represent the current situation in the country, especially with changes in the epidemiology of HIV whose prevalence has gone down from more than 15%^14^ to about 8.7% with 85% of these receiving ART^18^. The health and economic challenges faced by the country with respect to public health priorities, clinical and laboratory expertise, the inadequacy of financial resources militate against the early diagnosis and treatment of fungal infections. Consequently, these health system limitations contribute to high rates of morbidity and mortality^105^.

As most serious fungal infections are opportunistic infections, a majority of the affected individuals are immunocompromised^4^. For example, cryptococcal meningitis, oesophageal candidiasis, PCP as well as aspergillosis are among the most common systemic fungal infections observed in HIV and AIDS patients, thus a combination of the underlying immunocompromised and superimposed fungal infection contributes to a higher risk of mortality^106^. The comorbidities necessitate the co-administration of drugs, resulting in drug-drug interactions (DDIs). In some cases, optimum therapy for fungal infections is contraindicated in conjunction with medicines used to treat co-morbid conditions in an attempt to prevent potential adverse effects and treatment failure^107,108^.

The preferred treatment for these fungal infections includes the administration of amphotericin B or azole antifungals such as fluconazole, itraconazole and voriconazole. All of these except voriconazole are on Zimbabwe’s essential list of medicines^109^ and studies from other countries have suggested that renal function should be closely monitored with concomitant use of amphotericin B and tenofovir as both drugs can cause nephrotoxicity. Similarly, combination therapy of zidovudine and amphotericin B may result in anaemia and neutropenia^108^. Hence, as DDIs are often unavoidable in HIV-infected patients, the potential effects of these DDIs cannot be ignored^106^ especially in Africa where very few drugs used have been evaluated for DDIs and pharmacogenetics^110^. This will potentially help in the management of patients suffering from co-morbidities as well as broaden our understanding of the effect of these DDIs in different populations. In addition to DDIs there is also a possibility of antifungal resistance^111^. Previous studies have reported fluconazole resistance among *C. neoformans* complex isolates from Africa^112^ and among *Candida* spp. isolated from women with VVC^113^. This has a large impact on health and well-being of affected individuals.

### Study limitations

Despite an exhaustive search in this study, we could not obtain enough local data to use for a precise estimate of the current burden, as most of the available studies were outdated. So, majority of the data used was obtained from other countries, which may introduce some inaccuracies when estimating the burden in Zimbabwe due to socioeconomic and geographical differences^114^. Another significant limitation is the incomplete nature of the estimates: for example, we could not estimate the burden of PCP in children or non-HIV patients, the burden of mycetoma, chromoblastomycosis as well sporotrichosis could not be estimated due to paucity of data despite reports of cases in the country. Nonetheless, our results show that fungal diseases are probably much more common than are documented in clinical practice. Our estimates provide a starting point from which to better understand the extent of the problem in the country and create awareness and propose appropriate studies and interventions to address fungal diseases that are of significant public health importance.

### Recommendations

Educating the community about fungal diseases is an important step in raising awareness about the morbidity and mortality associated with these diseases. For most villages and communities across Zimbabwe, the entry point to health and health information dissemination is vested in the Community/Village Health Workers. These people are therefore integral in promoting awareness and assisting in early detection of symptoms associated with fungal diseases. Their knowledge of a community’s languages and customs means that they are able to deliver health messages to groups in a culturally appropriate manner, which is also easily understood. This is an effective means of disseminating information to community members resulting in community-based surveillance, which improves the likelihood of early case detection^115^, as well as reducing the stigma associated with some of the fungal diseases. The medical mycology community in Zimbabwe should therefore work closely with local organisations and community health workers to raise awareness of fungal diseases.

Schools are also a good place to promote campaigns aimed at raising awareness about fungal infections. For example, the neglected tropical disease, schistosomiasis, is a public health burden in Zimbabwe^116^. Educational campaigns in schools including essay competitions, drama and ‘edutainment’ are ways in which awareness about the disease is raised in communities. A similar approach could be used to create awareness about fungi diseases among school children.

To increase the access to better diagnostics locally, a standardised diagnostic algorithm based on clinical signs and symptoms that can be easily identified by primary healthcare professionals^117^ can be developed. The algorithm will help in early diagnosis and treatment of fungal diseases as well as ensure wealth of information on fungal diseases affecting the African population. This will allow interventions to be implemented in the primary healthcare setting and has the potential to significantly reduce morbidity and improve quality of life.

## Conclusion

This study is the first to estimate the burden of fungal diseases in Zimbabwe and to provide an estimation of its impact on public health. The paucity of data on fungal infections in the country warrants for further epidemiology studies and better diagnostics to aid patient management.

## Methods

The prevalence and incidence of fungal disease in Zimbabwe were calculated following methods previously described^118,119^ and the applied formulae will be detailed below. The burden was estimated for the general healthy population and for the ‘at- risk’ populations including HIV/AIDS patients, survivors of pulmonary TB, cancer, chronic obstructive pulmonary disease (COPD), asthma and patients receiving critical care. National or local data were preferred, but where these were unavailable, data were extrapolated from other sources. The annual burden was estimated for each fungal disease and presented as: (i) absolute number of cases per year in the country and (ii) annual rates. The absolute cases were presented as either incidence or prevalence depending on the nature of infection. The annual rates (incidence or prevalence) were calculated using the absolute annual number of cases as the numerator and the entire Zimbabwean population as the denominator. For simplicity, the 2019 Zimbabwean population (n = 14,863,000^120^) was used regardless of the year from which the numerator data originated. The United Nations population estimates 2019, WHO reports, and The Joint Nations Programme on HIV/AIDS (UNAIDS) were used for the population demographics.

### Calculating fungal disease burden

Prevalence or incidence was calculated using data from published studies. Prevalence was calculated for allergic bronchopulmonary aspergillosis (ABPA), severe asthma with fungal sensitisation (SAFS), chronic pulmonary aspergillosis (CPA), recurrent vulvovaginal candidiasis (RVCC), and tinea capitis and the remaining estimates were calculated as annual incidence. Due to paucity of data, we were not able to calculate both prevalence and incidence for each disease.

### Prevalence

To calculate the prevalence of ABPA, SAFS, CPA, RVCC, and tinea capitis we applied the same formulae used by GAFFI members to estimate the prevalence of fungal diseases in other countries^55,60,121,122^.

### Annual incidence

To calculate the incidence of invasive aspergillosis, oesophageal candidiasis, candidemia and *Candida* peritonitis we applied the same formulae used to estimate the incidence in other countries^55,60,121,122^. For cryptococcal meningitis, *Pneumocystis* pneumonia and oral candidiasis, instead of calculating a figure based on the denominator, we doubled our at-risk population (ART naïve), being cognisant of the fact that virological failure does not always translate to immunodeficiency.

$${\mathbf{Cryptococcal}}\,{\text{ }}{\mathbf{meningitis}} = {\text{ }}\left( {{\text{Annual }}\,{\text{new }}\,{\text{AIDS}}\,{\text{ cases}} \times {\text{Proportion }}\,{\text{of }}\,{\text{AIDS}}\,{\text{ patients }}\,{\text{presenting }}\,{\text{with }}\,{\text{cryptococcal }}\,{\text{meningitis}}} \right){\text{ }} \times {\text{2}}$$

$$Pneumocystis\,{\mathbf{pneumonia}} = {\text{ }}\left( {{\text{Annual }}\,{\text{new}}\,{\text{ AIDS }}\,{\text{cases}} \times {\text{Proportion }}\,{\text{of }}\,{\text{AIDS}}\,{\text{ patients }}\,{\text{presenting }}\,{\text{with}}\,Pneumocystis\,{\text{pneumonia}}} \right){\text{ }} \times {\text{2}}$$

$${\mathbf{Oral}}\,{\text{ }}{\mathbf{candidiasis}} = {\text{ }}\left( {{\text{Annual }}\,{\text{new }}\,{\text{AIDS cases }} \times 0.{\text{9}}} \right){\text{ }} \times {\text{2}}$$

The values above and the assumptions made to obtain the accurate denominators were obtained from a systematic literature search detailed below. The assessment of the quality of the source data, country profile and assumptions made for the analyses are detailed below.

### Data sources and search terms

Published papers were identified from four databases: PubMed, Web of Science, EMBASE and Google Scholar. The following search terms were used: fungal infection, fungal burden, fungal epidemiology, Zimbabwe, Southern Africa, and Africa. A second search included the same searches using the following diseases: *Cryptococcus*/cryptococcal, *Candida*/thrush, *Aspergillus*/aspergillosis, histoplasmosis, asthma, leukaemia, chronic obstructive pulmonary disease (COPD), *Pneumocystis* pneumonia/*Pneumocystis jirovecii* pneumonia (PJP)/*Pneumocystis carinii* pneumonia, chronic pulmonary aspergillosis (CPA), aspergilloma, allergic bronchopulmonary aspergillosis (ABPA), severe asthma with fungal sensitisation (SAFS), tinea/ringworm. We used HIV data to estimate the burdens of cryptococcal meningitis (CM), candidiasis and *Pneumocystis jirovecii* pneumonia (PCP). Asthma, chronic obstructive pulmonary disease and tuberculosis data were used to estimate the presumed burden of allergic bronchopulmonary aspergillosis (ABPA) and chronic pulmonary aspergillosis (CPA). Burdens of candidaemia and *Candida* peritonitis were derived from critical care and/ or cancer patients’ data.

Papers presenting incidence or prevalence of any fungal disease were evaluated using an adapted Grading of Recommendations, Assessment, Development and Evaluations (GRADE) score^123^ based on the following features: diagnostic accuracy, study size (using a cut-off of > 10 cases), year of study, with more recent studies scoring higher, type of publication, with original research article scoring more, methodology and country, with studies from Zimbabwe scoring higher (Table 2). Those with an adapted GRADE mean score of > 2 were deemed acceptable and enabled a minimum estimation of the country burden of fungal diseases (Table 3). Papers with a mean score < 2 were excluded in the estimation of the country’s burden of fungal diseases but were discussed in the review.

**Table 2:** Scoring system for modified GRADE criteria.

Diagnostic	Score
PCR + laboratory + clinical + imaging	2
Culture, smear, histology	1
Clinical suspicion only	0
**Patient sample size**	Score
≥ 10	1
< 10	0
**Year of study**	Score
< 5 years	2
5–10 years	1
> 10 years	0
**Country (data used)**	Score
Zimbabwe	2
Any other African country	1
Rest of the world	0
**Methodology (well designed)**	Score
Yes	1
No	0
**Type of publication**	Score
Research paper	2
Case study/short reports	1
Review papers	0
**Possible total score**	**10**

**Table 3:** Modified GRADE score for the papers used for estimating burden of fungal diseases in Zimbabwe.

Disease	Diagnostic accuracy	Patient sample size n > 10	Up to date	Type of publication	Methodology	Country	Overall score	References
PCP	2	1	1	2	1	1	8	^24^
Histoplasmosis	1	1	1	1	0	2	6	^10^
Invasive aspergillosis	2	1	1	2	1	0	6	^124^
	1	1	0	1	1	0	4	^125^
	1	1	1	1	1	0	5	^126^
	0	1	1	1	1	0	4	^127^
Candidaemia	–	–	1	0	1	–	2	^128^
*Candida* peritonitis	1	1	1	1	1	0	5	^129^
ABPA	–	–	1	0	1	–	2	^130^
SAFS	–	–	1	0	1	–	2	^89^
CM	–	–	2	0	1	–	3	^131^
RVVC	–	–	2	0	1	0	3	^132^
Tinea capitis	1	1	0	2	1	2	7	^9^
Mucormycosis	0	1	1	1	1	0	4	^133^
Fungal keratitis	–	–	1	0	1	–	2	^34^

*CM* Cryptococcal meningitis, *PCP* Pneumocystis pneumonia, *CPA* chronic pulmonary aspergillosis, *ABPA* allergic bronchopulmonary aspergillosis, *SAFS* severe asthma with fungal sensitisation, *IA* invasive candidiasis, *RVVC* recurrent vulvovaginal candidiasis.

### Country profile

Zimbabwe is a landlocked country situated in Southern Africa, between the Zambezi and Limpopo Rivers, bordered by Botswana, Mozambique, South Africa and Zambia. In 2019, the Zimbabwean population was projected to be 14.9 million, with 58% adults^120^. The number of people living with HIV/AIDS (PLWH) as of 2019 was estimated to be 1.3 million. The population estimates and HIV-related deaths were obtained from World Population Prospects and UNAIDS respectively and are shown in Table 4^18,120^. National TB data were obtained from the World Health Organization (WHO)^80^. National prevalence data for lung cancer, chronic obstructive pulmonary disease (COPD), diabetes and incidence data for acute myeloid leukemia (AML) were obtained from the 2016 Global Burden of Disease study^134^. To estimate the burden for HIV-related fungal diseases, we have assumed a 7-year linear decline in CD4 count to < 200 × 10^6^/l, of those not on ART, doubled to reflect those on ART who fail with ARV resistance or default (at risk of opportunistic infections).

**Table 4:** Country’s profile. Populations and rates required to calculate burden fungal-related diseases in Zimbabwe.

		Patient numbers and rates	Source
Demographics	Total population	14,863,000	^120^
	Children (< 15 years),	6,230,000	
	Total number of adults,	8,633,000	
	Adult women	4,489,160	
HIV/AIDS	Current total HIV/AIDS	1,400,000	^18^
	Children with HIV	84,000	
	Proportion of diagnosed cases on ARVs	85%	
	Number of diagnosed cases receiving ARVs	1,100,000	
	Proportion of those on ARVs who fail or have ARV resistance Number of diagnosed cases not receiving ARVs	11% 300,000	^135^
	Annual new AIDS cases (at risk of OIs)	42,857	
	AIDS-related deaths	20,000	
Respiratory diseases	Pulmonary tuberculosis annual incidence (survivors)	20,430	^80^ ^28^ ^136^ ^137,138^
	Prevalence of asthma in adults	6.9%	
	COPD prevalence (all GOLD stages) COPD hospital admissions	7.8% 121,728	
Lung cancer		744	^134^
Diabetes		4.6%	^139^
Leukaemia	AML	230	^140^

*COPD* chronic obstructive pulmonary disease, *GOLD* Global initiative for Obstructive Lung Disease, *ARV* antiretroviral, *OI* opportunistic infection, *AML* acute myeloid leukaemia.

Assumptions from other published reports were used to identify the most accurate denominators to use for our estimates and these are summarised in Table 5**.** In brief, PCP frequency was estimated by assuming 11% of newly diagnosed HIV/AIDS adults with the risk spread over 2 years^24^. The prevalence of AIDS patients presenting with cryptococcal meningitis was assumed to be 7.1% based on a study by Rajasingham et al.^131^ among antiretroviral therapy (ART)-naive HIV patients. Chronic pulmonary aspergillosis (CPA) prevalence was estimated using the previously described approach taken by Denning et al*.*^141^, where the number of annual PTB cases with cavities (22%) was multiplied by the incidence of CPA in cavities (22%) and the number of PTB cases without cavities (78%) was multiplied by CPA incidence (2%). An estimation of a 5-year prevalence of CPA was made, assuming a 15% annual mortality or surgical cure rate^130^. To calculate all cases of CPA, PTB was assumed to be the underlying disorder in 67% of cases^142^. Invasive aspergillosis was estimated in haematological and lung malignancies, HIV/AIDS and COPD. It was assumed that 10% of acute myeloid leukemia (AML) patients develop IA and that an equal number of cases are found in non-AML haematological patients while 1.3% of admitted COPD patients^124^, 2.6% of lung cancer patients^127^ and 4% HIV/AIDS patients who died develop IA^125^. ABPA estimation was made assuming that 2.5% of adult asthmatics have ABPA^130,143^ and although ABPA also occurs in cystic fibrosis, no estimate of the prevalence of this disease in Zimbabwe was attainable. The estimate of SAFS was as estimated at 33% of the most severe asthmatics (10%)^89^.

**Table 5:** Assumptions on which estimates of fungal diseases were made.

Fungal disease	Underlying condition	Assumptions made	References
Cryptococcal meningitis	HIV/AIDS	Assumes 7.1% of AIDS patients	^131^
*Pneumocystis* pneumonia	HIV/AIDS	Assumes 11% PCP as newly diagnosed HIV/AIDS adults over 2 years	^24^
Invasive aspergillosis	HIV/AIDS; COPD; Leukaemia; lung cancer	Assumes 10% of AML patients develop IA. Rate in non-AML same as in AML. 1.3% of admitted COPD patients, 2.6% of lung cancer patients and 4% of HIV/AIDS deaths	^124–127^
Chronic pulmonary aspergillosis	Tuberculosis, COPD	Assumed that 22% of those with and 2% of those without cavities after TB develop CPA; that pulmonary tuberculosis is the underlying diagnosis in 67% of all CPA cases	^141^
Allergic bronchopulmonary aspergillosis	Asthma	Assumed to occur in 2.5% of adult asthmatics	^28,130,143^
Severe asthma with fungal sensitisation	Severe asthma	Assumes 33% of worst 10% of adult asthmatics	^89^
Candidemia		5/100,000 (mean of 2-11/100,000) with 30% in ICU (critical care and post-surgical patients) and 70% in cancer and other immunocompromised patients	^128^
*Candida* peritonitis	Pancreatitis, major abdominal surgery	Assumes 1 patient with hospital-acquired (almost all post-operative) Candida peritonitis for every 2 patients with candidaemia, in ICU	^129^
Oral candidiasis	HIV/AIDS	Assumes it occurs in 90% of untreated HIV patients, over 2 years	^144^
Oesophageal candidiasis	HIV/AIDS	20% of patients not on ARVs, and 0.5% of those on ARVs	^145,146^
Recurrent Vulvovaginal Candidiasis (≥ 4x/year)		6% of adult women	^132^
Mucormycosis		Assumes that it affects 2 per million of the population based on data from Europe	^133^
Histoplasmosis	HIV/AIDS	Based on literature	^10^
Tinea capitis		Assumes 29%, based on a study by Robertson and Wright (1990)	^9^

*COPD* chronic obstructive pulmonary disease, *CPA* chronic pulmonary aspergillosis.

Oral candidiasis was assumed to affect 90% of untreated HIV patients over 12 months, based on a study in Tanzania^144^. Oesophageal candidiasis was assumed to affect 20% of advanced HIV disease patients and 0.5% of HIV patients on ARV treatment^145,146^. Mucormycosis was estimated to occur at a rate of 0.2/100,000 (literature estimate)^25^. Candidaemia cases were estimated assuming it occurs at a rate of 5 per 100,000 with 30% in ICU (critical care and post-surgical patients) and 70% in cancer and other immunocompromised and hospitalised patients^128^. For *Candida* peritonitis (intrabdominal candidiasis), we assumed that the rate was half of the ICU candidemia rate^129^. The estimated prevalence of RVVC was established assuming a frequency rate of 6% in adult women^132^. Tinea capitis was estimated at 29% prevalence amongst schoolchildren in Zimbabwe^9^.
